# Comparison of Autologous Blood Clots with Fibrin Sealant as Scaffolds for Promoting Human Muscle-Derived Stem Cell-Mediated Bone Regeneration

**DOI:** 10.3390/biomedicines9080983

**Published:** 2021-08-09

**Authors:** Xueqin Gao, Haizi Cheng, Xuying Sun, Aiping Lu, Joseph J. Ruzbarsky, Bing Wang, Johnny Huard

**Affiliations:** 1 Center for Regenerative Sports Medicine, Steadman Philippon Research Institute, Vail, CO 81657, USA; xgao@sprivail.org (X.G.); alu@sprivail.org (A.L.); 2 Department of Orthopaedic Surgery, McGovern Medical School, University of Texas Health Science Center at Houston, Houston, TX 77054, USA; Haizi.Cheng@uth.tmc.edu (H.C.); sunxuying1984@hotmail.com (X.S.); 3 The Steadman Clinic, Vail, CO 81657, USA; jruzbarsky@thesteadmanclinic.com; 4 Vascular Medicine Institute, University of Pittsburgh, Pittsburgh, PA 15140, USA; bingwang@pitt.edu; 5 Department of Medicine, Division of Cardiology, School of Medicine, University of Pittsburgh, Pittsburgh, PA 15140, USA; 6 McGowan Institute for Regenerative Medicine, University of Pittsburgh, Pittsburgh, PA 15140, USA

**Keywords:** blood clot scaffold, fibrin sealant, human muscle derived stem cells, lenti-BMP2/GFP, bone regeneration, bone tissue engineering

## Abstract

**Background**. Fibrin sealant has been used as a scaffold to deliver genetically modified human muscle-derived stem cells (hMDSCs) for bone regeneration. Alternatively, autologous blood clots are safe, economic scaffolds. This study compared autologous blood clot (BC) with fibrin sealant (FS) as a scaffold to deliver lenti-BMP2/GFP-transduced hMDSCs for bone regeneration. **Methods**. In vitro osteogenic differentiation was performed using 3D pellet culture and evaluated using microCT and Von Kossa staining. The lenti-GFP transduced cells were then mixed with human blood for evaluation of osteogenic differentiation. Furthermore, a murine critical- sized calvarial defect model was utilized to compare BC and FS scaffolds for lenti-BMP2/GFP-transduced hMDSCs mediated bone regeneration and evaluated with micro-CT and histology. **Results**. Lenti-BMP2/GFP transduced hMDSCs formed significantly larger mineralized pellets than non-transduced hMDSCs. hMDSCs within the human blood clot migrated out and differentiated into ALP^+^ osteoblasts. In vivo, BC resulted in significantly less new bone formation within a critical-sized calvarial bone defect than FS scaffold, despite no difference observed for GFP^+^ donor cells, osteoclasts, and osteoblasts in the newly formed bone. **Conclusions**. Human lenti-BMP2/GFP-transduced hMDSCs can efficiently undergo osteogenic differentiation in vitro. Unexpectedly, the newly regenerated bone in BC group was significantly less than the FS group. The autologous blood clot scaffold is less efficacious for delivering stem cells for bone regeneration than fibrin sealant.

## 1. Introduction

Treatment of fracture non-unions and segmental bone defects are challenging clinical scenarios. Bone tissue engineering mimics bone healing by providing bone morphogenic proteins, stem cells and a supportive or bioactive scaffold. Many osteo-inductive scaffolds, such as demineralized bone matrix, biphasic calcium phosphate [1,2,3,4,5], tricalcium phosphate (TCP) [6,7], hydroxyapatite [8], and collagen sponge or gel [9,10], have been used as supportive materials for bone regeneration. Previous work by our group compared different scaffolds for the use of delivery of muscle derived stem cell-mediated bone regeneration. The results demonstrated that fibrin sealant (Tisseel, Baxter)-mediated bone regeneration led to a bone tissue quality comparable to native bone [11]. Furthermore, fibrin sealant has also been used as a scaffold for bone regeneration in critical-sized calvarial bone defects to address different questions of bone tissue engineering, such as gene function and aging [12,13,14,15,16,17,18]. The utilization of fibrin sealant as a scaffold has several advantages, as it is a natural blood clot product, as well as safe and fully absorbable by day 7 after implantation [14]. A fibrin sealant scaffold, in essence, mimics natural blood clot formation with fibrin and thrombin but without other blood components. It is known that the blood clot or hematoma formation is the first stage of callus formation, initiating bone fracture or defect healing as well as other healing tissue injuries [19]. More than two decades ago, it was shown that utilizing exogenous blood clots for meniscal repair enhanced healing by providing chemotactic and mitogenic stimuli for reparative cells [20]. More recently, human blood clots were found to have the ability to preserve human stem cells within the clot, further drawing interest in its utility as a potential biologic scaffold [21].

The use of blood clots for different types of tissue regeneration applications have also proven effective for bone regeneration. In a rat osteotomy model, autologous blood clots loaded with bone morphogenetic protein-2 (BMP2) demonstrated the ability to completely bridge the defect, while in situ hematoma and artificial blood clot groups did not bridge the defect [22]. These results indicate that autologous blood clots can be a suitable scaffold for delivery of BMPs, but BMP supplementation is usually required [22]. Furthermore, blood clots in combination with biphasic calcium phosphate (BCP) alone demonstrated the ability to repair a rat femoral defect even without the presence of BMPs [23]. The OSTEOGROW device, which utilizes bone morphogenetic protein 6 (BMP6) and a whole blood coagulum, has been observed to promote bone formation without increasing bone resorption [24]. Based on these promising results of autologous blood clots as a scaffold for bone regeneration, in this study, it is hypothesized that autologous blood clots can serve as both a scaffold and simultaneously provide nutrition for transplanted stem cells until host circulation is re-established in a bone defect. Furthermore, blood clots have promising potential to support human muscle-derived stem cell (hMDSC) survival and osteogenic differentiation and improve qualitative and quantitative bone formation when compared to fibrin sealant.

## 2. Materials and Methods

### 2.1. Cell Isolation and Lenti-BMP2/GFP Construction

Human muscle-derived stem cells (hMDSCs) were isolated from a 74-year-old male using the preplate technique [25]. The preplate 6 (PP6) cells, also called hMDSCs, have been proven to express high levels of mesenchymal stem cell markers and exhibit multipotent differentiation potential [13]. The lenti-BMP2/GFP viral vector was constructed by Dr. Bing Wang at the University of Pittsburgh. The hMDSCs were transduced with lenti-BMP2/GFP viral vector at a dilution of 1:8 with overnight incubation using polybrene (8 µg/mL). Lenti-BMP2/GFP-transduced hMDSCs were then subjected to fluorescence-activated cell sorting for isolating GFP-positive transduced cells. Secretion of BMP2 in the culture medium was measured with BMP-2 Quantikine ELISA Kit (DBP200, R&D system, Minneapolis, MN, USA).

### 2.2. In Vitro Osteogenesis Using 3D Pellet Culture

Lenti-BMP2GFP-transduced hMDSCs and non-transduced hMDSCs were expanded in proliferation medium containing DMEM high glucose supplemented with 20% fetal bovine serum (FBS), 1% chicken embryo extract, and 1% penicillin/streptomycin. Pellet cultures were performed using a protocol previously described [13,15,17]. Briefly, 2.5 × 10^5^ cells were aliquoted into 15 mL conical tubes and centrifuged at 2000 rpm for 5 min, after which the supernatant was removed. The cells were then resuspended with osteogenic medium containing DMEM high glucose supplemented with 10% FBS, 10^−2^ M sodium glycerophosphate, 50 μg/mL ascorbic acid, and 10^−7^ M dexamethasone (all from Sigma-Aldrich, St. Louis, MO, USA). The cells were mixed and centrifuged at 500× *g* for 5 min. The pellet was then cultured in osteogenic medium for 28 days and the osteogenic medium was changed every 2–3 days. After 28 days, pellets were fixed in neutral buffered formalin (NBF) for 1 h and scanned with microCT to detect mineralization using 70 kVP and 114 µA with 25 µm resolution (voxel size) using Viva CT 40 (Scanco Medical AG, Fabrikweg 2, Brüttisellen, Switzerland). After the micro-CT scan, the pellets were embedded in NEG frozen medium and flash frozen in liquid nitrogen. Next, 8 µm sections were cut and Von Kossa staining was performed using the IHC world protocol (Woodstock, MD, USA).

### 2.3. Cell Survival in Blood Clot

In order to test whether the blood clot would maintain cell survival and osteogenic differentiation capacity, 10 mL of fresh drawn human blood (Institutional Review Board of University of Texas Health Science Center at Houston approved as part of previous study [21]) was mixed with 5 × 10^5^ cells of lenti-GFP-transduced hMDSCs in 1 mL phosphate buffered saline (PBS). After the clot formed, the blood clot was wrapped with a sterile woven cotton sponge in order to remove any residual blood. The blood clots were then cut into six segments and cultured at 37 °C and 5% carbon dioxide (CO_2_) in 12 well plates for 7 days. Following this, the blood clots were removed from the plate wells. Then, the adhered cells that migrated out of the blood clot were replaced with fresh medium and imaged for GFP using a fluorescent microscope. These same cells were then further cultured in osteogenic medium or proliferation medium (three wells each) for 25 days. The cells were then fixed in NBF for 2 min and stained for the osteogenic marker, alkaline phosphatase, to validate osteogenic differentiation following the manufacturer’s protocol (86C Kit, Sigma-Aldrich, St. Louis, MO, USA).

### 2.4. Optimization of the Volume of Whole Blood and Cells for Clot Formation

PBS was tested for its potential effect on blood clot formation time, as it is necessary for cells to be resuspended in PBS prior to implantation. Two approaches of drawing blood from mice were also tested in addition to different volumes of blood mixed with PBS.

Test 1: Draw blood from mice heart, and test in the 1.5 mL Eppendorf tube:

(1) 40 µL blood + 20 µL PBS;
(2) 45 µL blood + 15 µL PBS;
(3) 50 µL blood + 10 µL PBS;
(4) 50 µL blood only.

Test 2: Clot formation in the skull defect environment

First, a critical-sized 5 mm calvarial skull defect was created, after which blood was drawn from the retro-orbital space of mice using a sterile capillary glass pipet. The blood was then deposited from the capillary tube and placed onto a petri dish. Different volumes were then measured and mixed with different volumes of PBS (options #1 through #3 below), which were then placed into the critical-sized calvarial bone defect with a custom cut 200 µL pipet tip:

(1) Add blood 50 µL to 10 µL PBS
(2) Add blood 50 µL to 20 µL PBS;
(3) Add 50 µL blood to the defect.

Blood clot formation time was documented for each condition.

### 2.5. Creation of a 5-mm Critical-Sized Calvarial Defect

The in vivo animal experiment was approved by Animal Welfare Committee (AWC-15-0074) of the University of Texas Health Science Center at Houston. Male ICR-SCID mice (Taconic Biosciences, Rensselaer, NY, USA) were divided into two groups (*n* = 6); a fibrin sealant scaffold (FS) group, and a blood clot (BC) group. A 5-mm critical- sized calvarial bone defect was created in each mouse using a trephine drill, as previously described [13,15,16,17]. Briefly, after sterilization of the skin of scalp, an incision was made just off the midline, exposing the right parietal bone, and after removing the periosteum with scalpel, a 5 mm bone defect was created in the right parietal bone using 5 mm drill trephine. Throughout drilling, saline irrigation was performed to prevent overheating and potential cell necrosis during drilling process. Care was taken to prevent damage of both the dura mater and the brain tissue. Then, the cells and scaffold were implanted into the defect according to the steps described in Section 2.6.

### 2.6. Application of Lenti-BMP2/GFP-Transduced hMDSCs Plus Scaffold

For the FS group, after creation of the defect, 1.5 × 10^6^ lenti-BMP2/GFP-transduced hMDSCs (20 µL) were mixed with 25 µL of thrombin and added to the defect area using custom cut 200 µL pipet tip rings to maintain cells in the defect area. Then, 25 µL of fibrin was added on top of the cells, which was allowed to set for approximately 1 min for fibrin gel formation. For the BC group, after creation of the defect, whole blood (approximately 100 µL) was collected in a sterile petri-dish drop-wise via retro-orbital plexus puncture using a sterile capillary tube. Immediately, 50 µL of blood was mixed with 1.5 × 10^6^ lenti-BMP2/GFP-transduced hMDSCs in 20 µL PBS and then added to the defect area in a custom cut 200 µL pipet tip ring. The blood/cell mixture was allowed to set for approximately 5 min to form a clot in a cylinder-like shape that covered the defect area. Following scaffold/cell implantation, each defect was then closed using three 4-0-prolene sutures.

### 2.7. MicroCT Scanning and Analysis

After surgery, mice were subjected to microCT scanning at days 1, 14, 28, and 42 post-surgery. The scanning parameters were 70 kVP and 114 µA using 30 µm resolution (voxel size) and live scanning under 2% isoflurane anesthesia. For microCT analysis, the overview of defect healing was analyzed by one observer (XG) using 400 × 200 dimension and sigma = 1, Gauss support = 0.8, and threshold = 163 using microCT evaluation program V6.6. To quantify the newly regenerated bone volume in the defect area, the new bone formed within the 212 axial microCT slices that spanned the defect area were carefully contoured for the views of interest. The contoured slices were then quantified automatically using a 3D evaluation program and applying the same thresholds as the overview analysis (Supplemental Figure S1). The software places a box to cover all of the contoured area to calculate the total volume, bone volume, and other bone microarchitectural parameters. New bone volume and the bone volume density of the regenerated bone were compared between the two groups.

### 2.8. Histology

Mice were sacrificed at day 42 after microCT scanning and the entire skull tissue was harvested and fixed in neutral buffered formalin for 3 days. The tissues were then decalcified with 10% *w*/*v* EDTA plus 1% sodium hydroxide for 1 month, and then processed for paraffin sectioning (5 µm sections). H&E was performed using Hematoxylin and Eosin (Sigma-Aldrich, St. Louis, MO 68178, USA) Herovici’s staining was used for general morphology and identification of collagen type 1 and 3 according to the literature [15,26]. Tartrate resistant acid phosphatase (TRAP) staining was performed using a Sigma 387A-1 KT (Sigma Aldrich, St. Louis, MO, USA) following the manufacturer’s protocol. Microscope images were captured using Nikon CIL upright microscope (Nikon Instruments Inc., Melville, NY, USA). TRAP^+^ cells were quantified using Image J and bone area and surface area was measured using Image J (ij153-win-Java 8) at the same time. TRAP^+^ cells/bone surface was calculated using Excel.

### 2.9. Immunohistochemistry of GFP and Osterix (OSX) 

Immunohistochemistry staining for GFP and OSX was performed as previously described [16,17]. Briefly, after deparaffinization the slides were washed with PBS three times and then blocked with 5% donkey serum in PBS for 1 h at room temperature. Sections were then incubated with primary antibody diluted in 5% donkey serum overnight at 4 °C. The primary antibody dilution was 1:1000 dilution for both GFP (Ab290, Abcam, Waltham, MA, USA) and OSX (ab22552, Abcam). Rabbit isotype control (I-1000-5, Vector Laboratories, Burlingame, CA, USA) was used as a negative staining control (1:1000 dilution). Slides were then washed with PBS and incubated with goat anti-rabbit IgG biotinylated secondary antibody (BA-1000, Vector Laboratories) for 2 h at room temperature. After another PBS wash, the slides were incubated for 2 h in VECTASTAIN^®^ Elite ABC-HRP Kit, Peroxidase (Standard) (PK-6100, Vector Laboratories) reagent with A and B premixed for 30 min. Slides were then washed with PBS and a color reaction was performed using a DAB kit (SK-4100, Vector Laboratories) for 6 min. Subsequently, slides were rinsed with tap water and nuclei were counterstained with Hematoxylin QS (H-3404-100, Vector Laboratories) for 30 s. Finally, slides were rinsed with running tap water for 10 min and dehydrated with gradient alcohol, cleared with xylene, dried, and cover glassed with Cytoseal mount medium (Fisher Scientific, Waltham, MA, USA).

### 2.10. Statistical Analysis

The unpaired two-tailed *t*-test was used to compare values between the two groups using GraphPad Prism 9 software. A *p* < 0.05 was considered statistically significant.

## 3. Results

### 3.1. hMDSCs Transduction Efficiency and BMP2 Secretion Levels

Following transduction, 65% of the cells were GFP-positive. The lenti-BMP2/GFP-transduced hMDSCs were sorted with GFP green fluorescence to enrich GFP^+^-transduced cells. The mean BMP2 secretion level by lenti-BMP2/GFP-transduced hMDSCs was 1.2 ± 0.25 ng/million cells/24 h after cell sorting for GFP.

### 3.2. Lenti-BMP2/GFP-Transduced hMDSCs Demonstrated Significantly Enhanced In Vitro Osteogenic Differentiation

**Figure 1:**
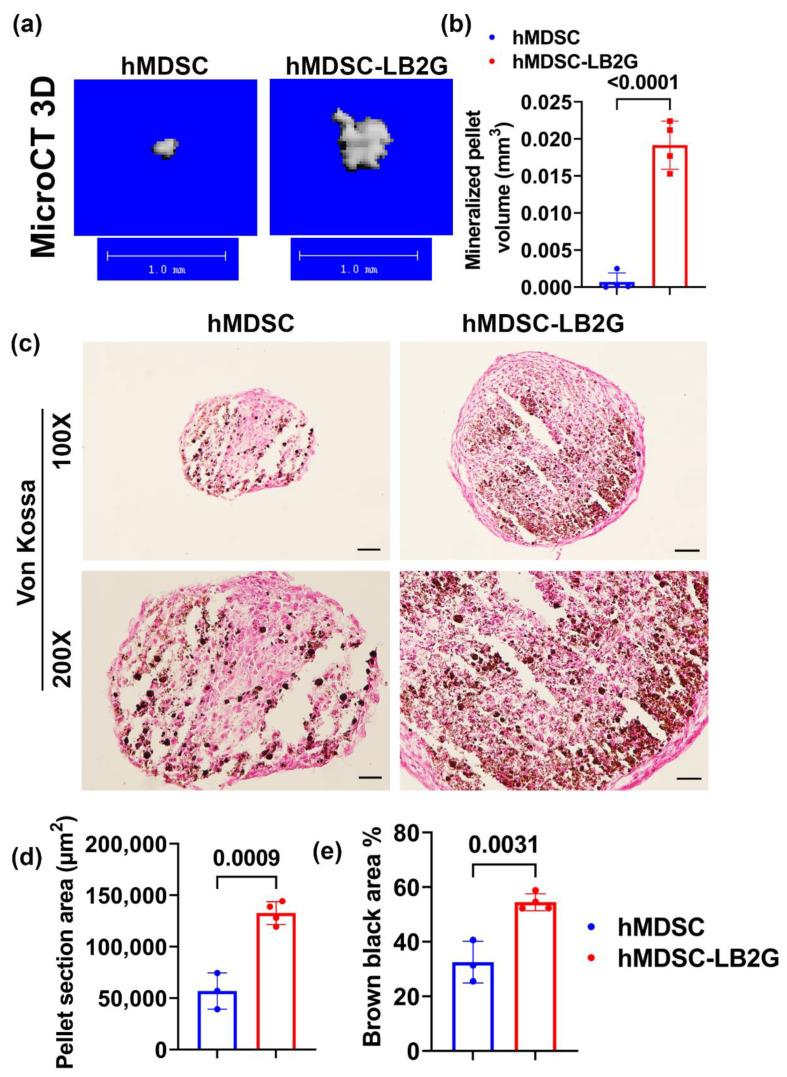
In vitro osteogenesis of lenti-BMP2/GFP-transduced hMDSCs compared to non-transduced hMDSCS. (**a**) MicroCT 3D images of osteogenic pellets at day 28. Lenti-BMP4/GFP-transduced hMDSC formed larger mineralized pellets. Scale bars = 1 mm. (**b**) Quantification of mineralized pellet volume. (**c**) Von Kossa staining showed bigger pellets and higher percentage of brown-black mineralization in lenti-BMP2/GFP-transduced hMDSCs than non-transduced hMDSCs. Scale bars = 100 µm for 100× images and 200 µm for 200× images. (**d**,**e**) Quantification of Von Kossa staining (100×) for pellet section area and brown-black calcium deposit percentage.

In vitro osteogenesis of lenti-BMP2/GFP-transduced hMDSCs was compared to non-transduced hMDSCS. MicroCT 3D results showed larger mineralized osteogenic pellets formed by lenti-BMP2/GFP-transduced cells when cultured for 28 days in osteogenic culture (Figure 1a). The mineralized pellet volume was significantly larger for lenti-BMP2/GFP-transduced hMDSCs than non-transduced hMDSCs (Figure 1b). Furthermore, Von Kossa staining showed significantly bigger pellets area and higher percentage of brown-black mineralization in lenti-BMP2/GFP-transduced hMDSCs than non-transduced hMDSCs (Figure 1c–e).

### 3.3. hMDSC Can Migrate out from Cultured Blood Clot and Undergo Osteogenic Differentiation

**Figure 2:**
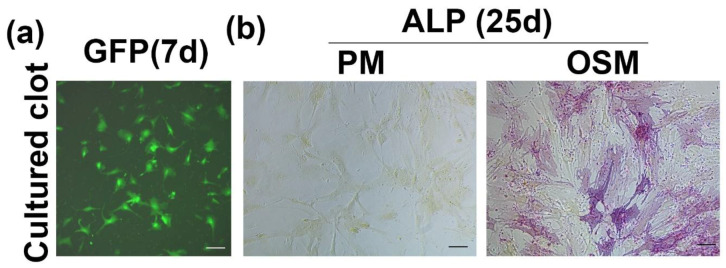
hMDSC can migrate out of the blood clot and undergo proliferation and osteogenic differentiation. (**a**) GFP^+^ hMDSCs migrated out from blood clot. Scale bar = 100 µm. (**b**) ALP staining of cells that migrated out from the blood clot cultured in proliferation medium (PM) and osteogenic medium for 25 days. Cells cultured in osteogenic medium express ALP, while cells cultured in PM (non-osteogenic) remain ALP-negative. Scale bar = 100 µm.

hMDSCs trapped in the blood clot during blood clot formation demonstrated the ability to migrate out from the blood clot as well as the ability to be grown in culture (Figure 2a). When the cells that migrated out of the blood clot were cultured in osteogenic medium for 25 days, these cells underwent osteogenic differentiation, as demonstrated by ALPstaining (Figure 2b).

### 3.4. Blood Clot Formed in the Skull Defect Injury Area Faster Than in the Plastic Tubes

To determine the optimal ratio of whole blood and cell suspension to ensure clot formation in vivo, several different conditions were tested. First, a 1.5 mL Eppendorf tube was tested. Surprisingly, the blood drawn from heart puncture did not form a clot in the tube after 20 min, even without the addition of PBS (Table 1, Test condition 1–4). Furthermore, the ratio was tested in vivo using the skull defect model. The blood was drawn from the same mice used for the skull defect surgery using the retro-orbital capillary method. Both whole blood alone and 50 µL whole blood mixed with 10 µL or 20 µL PBS formed a clot within 5 min after implantation into the skull defect area covered with a custom cut 200 µL tip cylinder ring. The formed clot took the shape of ring cylinder in the defect (Table 1 Test condition 5–7). Therefore, 50 µL of blood mixed with 20 µL PBS was chosen for the in vivo experiment of cell implantation with scaffolds based upon this condition, as the mixed fibrin sealant in 50 µL and cells in 20 µL volume were typically used when performing critical-sized cavarial bone defects using the fibrin sealant as a scaffold.

### 3.5. hMDSCs Regenerated Significantly Less Bone Using an Autologous Blood Clot Scaffold When Compared to a FS Scaffold

**Figure 3:**
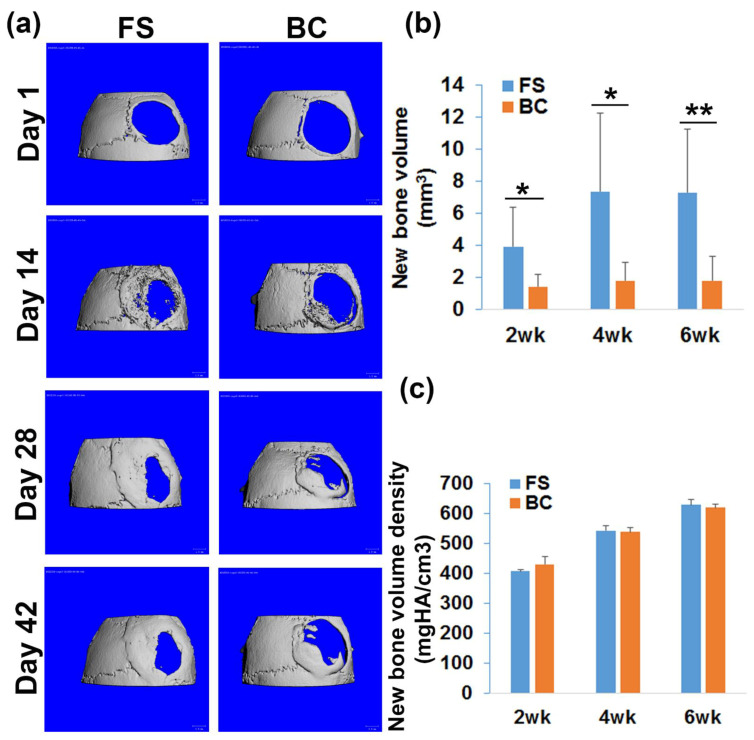
MicroCT analysis of bone regeneration. (**a**) MicroCT 3D images at different time points post-surgery. Scale bars = 1 mm. (**b**) Quantification of the new bone volume. There was significantly less new bone formed in the BC group than in the FS group. (**c**) New bone volume density quantification. Bone density increased as new bone matured. No statistically significant differences were found between the BC and FS groups. * *p* < 0.05, ** *p* < 0.01.

The feasibility of using autologous blood clot as a scaffold for hMDSC-mediated bone regeneration was tested and compared to an FS scaffold. One mouse in the FS group died after surgery and was excluded from the study due to the surgical complication. The microCT 3D images demonstrated bone regenerated in the defect area, but incomplete healing of the 5 mm critical-sized calvarial bone defect in both FS and BC groups at 6 weeks post-surgery (Figure 3a). Surprisingly, the newly regenerated bone volume in the BC group was significantly lower than that in the FS group at days 14, 28, and 42 post surgery (* *p* < 0.05, ** *p* < 0.01 compared to the FS group; Figure 3b). The bone defect healing percentage was 29.93% ± 16.51% and 22.5% ± 19.57% in the FS and BC groups, respectively. No statistically significant difference was observed between the groups. In addition, the new bone volume density of the regenerated bone was not significantly different between the FS and BC groups (Figure 3c).

### 3.6. Histological Analyses of the Regenerated Bone

**Figure 4:**
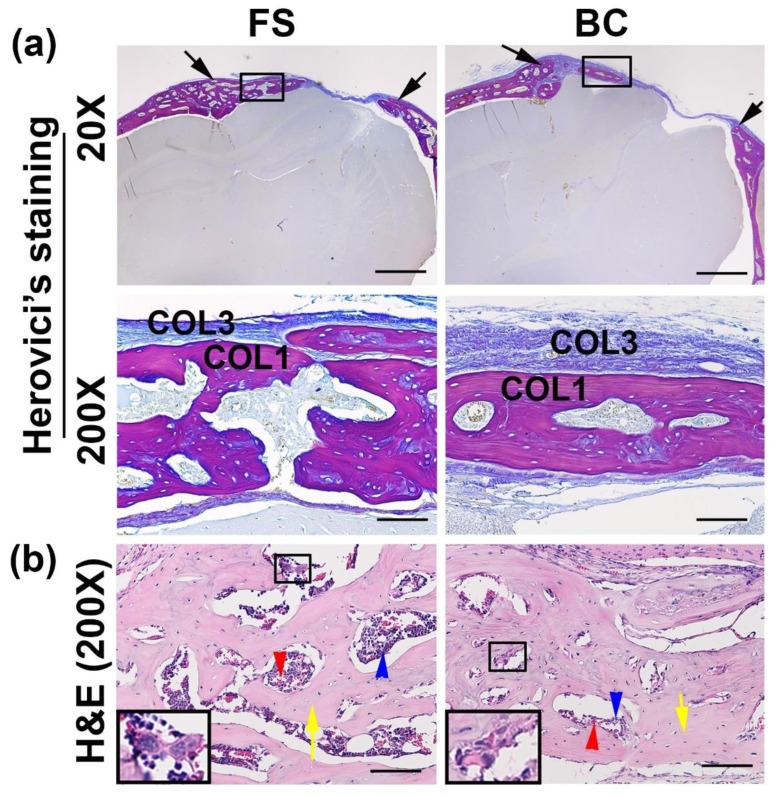
Histological analyses of the regenerated bone. (**a**) Herovici’s staining. The entire defect area is shown between the two black arrows in each image (top panels at 20×). Less collagen type I (red, COL1) matrix was found in the BC than in the FS group, in the defect area. Higher magnification (200×) showed the newly regenerated bone in the defect area (red, COL1). Fibrotic tissues are stained as blue fibers (COL3). (**b**) H&E staining showed that the newly regenerated bone was mainly trabecular bone and contained bone matrix (yellow arrows) and bone marrow that consisted of red blood cells (red arrows), hematopoietic cells (blue arrows), and megakaryocytes (insets). Scale bars = 1000 µm and 100 µm for 20× and 200× magnifications, respectively.

Herovici’s staining was performed to reveal the major bone structural protein, matrix collagen type 1 (COL1). The BC group formed less COL1 positive red matrix in the critical-sized calvarial bone defect area (Figure 4a). H&E staining showed the newly formed bone was functional trabecular bone that consisted of bone matrix (Figure 4b, yellow arrows) and bone marrow that contained red blood cells, hematopoietic cells, and megakaryocytes for both FS groups and BC groups (Figure 4b).

### 3.7. Participation of GFP-Positive Donor Cells and the Formation of Bone Cells in the Regenerated Bone Are Equivalent in the BC Group Compared to the FS Group

**Figure 5:**
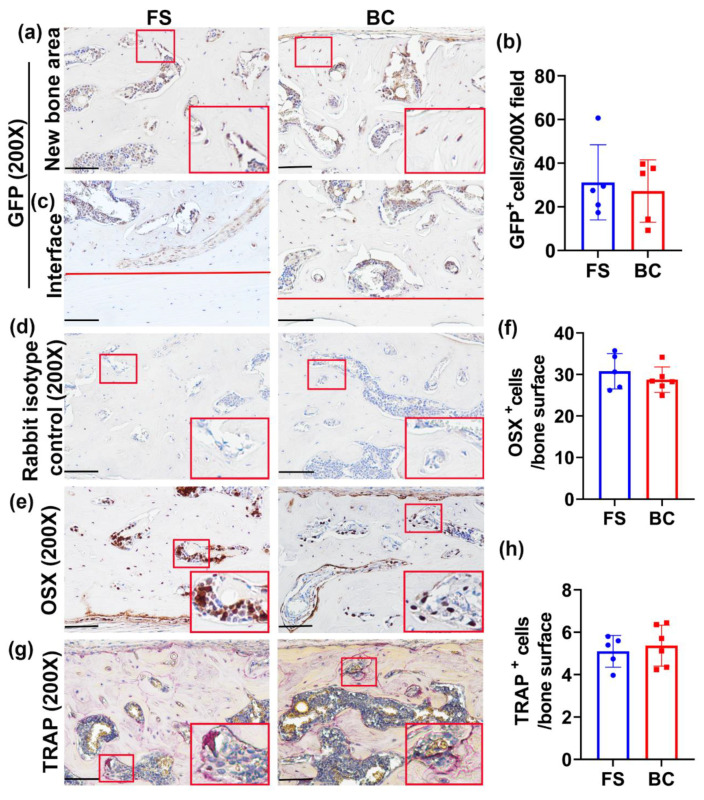
Histology staining of GFP donor cells and bone cells. (**a**) Immunohistochemistry of GFP for donor cells’ contribution to the regenerated bone. GFP^+^ cells showed in brown color on bone surface or in bone area. Few GFP^+^ cells were found in the bone surface (osteoblasts) and in the bone area (osteocytes). Insets are enlarged red box area to show GFP^+^ osteoblasts and osteocytes. Scale bars = 100 µm. (**b**) Quantification of GFP^+^ cells and normalized to cells/200× field. No statistical difference was found between the BC and FS groups. (**c**) GFP staining in the location of newly formed bone and the host–bone interface. The area above the red lines demonstrates new bone. GFP^+^ cells can be found on the bone surface or in the bone matrix for both groups in the newly formed bone area but not in the host bone area. Scale bars = 100 µm. (**d**) Rabbit isotype controls for both GFP and OSX staining of new bone area. All cells are negative. Scale bars = 100 µm. (**e**) Immunohistochemistry of OSX for osteogenic progenitor cells. OSX^+^ cells showed a brown color and were mainly located on the bone surface. Some osteocytes also expressed OSX. Insets are enlarged red boxed area to demonstrate OSX^+^ cells. Scale bars = 100 µm. (**f**) Quantification of OSX^+^ cells on the bone surface of newly regenerated bone. No statistical difference of OSX^+^ cells/bone surface was found between the BC and FS groups. (**g**) TRAP staining for osteoclasts. TRAP^+^ osteoclasts are seen in a violet-red color and exist as multinuclear or as single nuclear cells on the bone surface. Insets are enlarged red boxed area for TRAP^+^ cells. Scale bars = 100 µm. (**h**) Quantification of TRAP^+^ cells on the bone surface of the newly regenerated bone. No statistical difference was found between the BC and FS groups.

Immunohistochemistry of GFP was performed to determine the donor cells’ contribution to the regenerated bone. GFP^+^ cells were found in the bone surface (osteoblasts) and in bone area (osteocytes) (Figure 5a). There was no significant difference of GFP^+^ cells/bone area between the groups (Figure 5b). At the host bone and new bone interface, GFP^+^ cells were found in the new bone area, but not in the host bone area (Figure 5c). Rabbit isotype negative control showed negative staining (Figure 5d). Furthermore, immunohistochemistry of OSX for osteogenic progenitor cells was additionally performed. OSX^+^ cells were located on the bony surface, but some osteocytes also expressed OSX (Figure 5e). The OSX^+^ cells/bone surface demonstrated no statistical difference between the BC and FS groups (Figure 5f). In addition, TRAP staining for osteoclasts was used for detection of bone remodeling. TRAP^+^ cells (violet-red multinuclear or single nuclear) were detected on the bone surface of newly regenerated bone, a finding that is qualitatively similar to the host natural bone (data not shown) for both groups (Figure 5g). Quantification of TRAP^+^ cells/bone surface of the newly regenerated bone demonstrated no statistical differences between the BC and FS groups (Figure 5h). 

## 4. Discussion

This study demonstrates that lenti-BMP2/GFP hMDSCs undergo osteogenic differentiation more extensively than non-transduced hMDSCs. When the lenti-GFP-transduced hMDSCs are mixed in the blood clot, the cells survived and migrated out from the clot when cultured in hMDSCs proliferation medium. In addition, we found that lenti-GFP-transduced hMDSCs could further differentiate into ALP-positive osteoblasts when cultured in osteogenic medium. However, in vivo, autologous blood clot delivered lenti-BMP2/GFP-transduced hMDSCs regenerated significantly less bone than the delivery of the same number of cells in FDA-approved fibrin sealant, although, qualitatively, the regenerated bone was similar between the groups.

Both blood clot and fibrin sealant have been used as scaffolds for bone regeneration. Utilizing blood clots as scaffolds has gained more attention in recent years, given their autologous nature, safety profile, and lower cost, as well as their ability to circumvent the FDA approval process. It has been shown that autologous blood clots applied to a rabbit 2.7 mm non-critical-sized defect promotes bone formation when compared to hemostasis reagent microfibrillar collagen (8-fold increase), oxidized regenerated cellulose (1.5-fold increase), and equivalent to soluble alkylene oxide copolymer [27].The OSTEOGROW device, which utilizes a blood coagulum carrier to bind BMP6, has been found to stimulate mesenchymal stem cell differentiation and enhance healing of critical-sized ulnar bone defects without incurring bone resorption and inflammation. The device has demonstrated efficacy in regenerating bone in a canine bone defect healing model [24,28,29]. Furthermore, a recent study has shown that autologous blood clots can sustain-release BMP2 in vitro and promote osteogenic differentiation of stem cells. Autologous blood clot delivery of BMP2 at a 500 ng dose for a critical-sized murine calvarial defect or a 1 µg dose for a critical-sized rat calvarial defect significantly enhanced bone repair compared to a blood clot alone. Furthermore, blood clot scaffold-mediated bone regeneration can be further enhanced to aid in bone defect healing when illuminated with a near-infrared laser [30].

Blood clots have long been used as scaffolds in the oral cavity, where they have been utilized for tooth socket healing. Removal of the initially formed blood clot significantly delayed the socket healing, even when a new clot formed later on [31]. It has been shown that liposome-delivered BMP4 or TGF β1 were superior to PBS-delivered BMP4 and TGF β1 for tooth extraction socket healing when used alone or with a blood clot. Liposome-delivered BMP4 or TGF β1 increased the percentage of bone trabeculae and showed a higher number of blood vessels in the extracted tooth socket. Lower levels of immunoreactivity were also observed in the sockets filled with blood clots treated with liposomes only, PBS only, and BMP4 in PBS or TGF β1 in PBS when compared with sockets treated with liposome-delivered BMP4, TGF β1, or the combination of both in a rat model [32]. It has also been shown in a canine model that a blood clot combined with gel foam increased the ingrowth of hard tissues (2 out of 6 vs. 1 out of 4 and showed significantly more apical narrowing and hard tissue deposition when compared to scaffold-free controls [33].

Recent clinical trial results demonstrated that leukocyte- and platelet-rich fibrin-mediated defect filling showed the same dimensional and volumetric behavior as normal blood clots in healing the alveolar ridge in the setting of extracted teeth [34]. Blood clots have also been used for sinus subantral bone augmentation and resulted in less structural support than bone chips and hydroxyapatite [35]. Another study using a sinus augmentation model showed that venous blood clots did not induce enough bone formation to provide long-term support for sinus elevation [36]. Inflammatory factors and growth factors in the clot have the potential to be both beneficial and harmful. Specifically, granulocyte colony-stimulating factor (G-CSF) -enriched blood clots have been shown to have a negative effect on bone formation in a subcutaneous model [3].

On the other hand, fibrin such as commercialized fibrin (Tissucol), as a carrier, can sustain-release BMP2 for up to 28 days in vitro. In vivo, fibrin loaded with BMP-2 showed an extremely fast rate of bone healing, with a large amount of new bone formation throughout the entire defect in the first 4 weeks and complete cortical repair and fusion after 8 weeks, all with an absence of ectopic bone formation. In contrast, the control fibrin-only group did not fuse after 12 weeks, which indicates that fibrin itself does not have an osteo-inductive effect. Therefore, commercially available fibrin is a very efficient carrier for rhBMP-2 to promote critical-sized cortical bone defect healing and might be a more optimal delivery vehicle for BMP-2-induced bone regeneration than currently available collagen sponges [37].

Fibrin biopolymer (FBP), in combination with mesenchymal stem cells (MSCs), showed higher bone matrix deposition over time when compared with FBP plus biphasic calcium phosphate (BCP), MSCs with FBP in addition to BCP, and the FBP-only groups. BCP did not show additional benefits for bone formation [38]. However, it has also been shown that fibrin-mediated bone repair is negatively affected by the fibrinolysis inhibitor because it delayed fibrin matrix degradation [39]. It was found that if inhibitors were added, nondegraded matrix remained in the tissue even after 15 days and affected the migration of repair cells [39]. It is suggested that the use of aprotinin with fibrin glue may not be required, because liver, which is known to have high fibrinolytic activity, sealed and repaired well when using fibrin in the absence of plasminogen inhibitors [39].

Although both blood clots and fibrin have a very similar structure for the delivery of BMPs for bone regeneration, there is a paucity of direct comparisons for these two scaffolds in the setting of bone regeneration. In the current study, both the autologous blood clot and fibrin sealant were compared in parallel as delivery systems for lenti-BMP2/GFP-transduced hMDSCs. Our results indicated that blood clot delivery of lenti-BMP2/GFP-transduced hMDSCs resulted in significantly lower amounts of new bone formation within a critical-sized calvarial defect compared to the fibrin sealant. Since many studies showed that blood clots as scaffolds for bone regeneration without using cells are effective, it is possible that the blood clot is not suitable as a scaffold for delivery of live cells; rather, it is a more appropriate delivery vehicle for BMPs and other growth factors.

The reason why autologous blood clot scaffolds resulted in less bone formation than fibrin sealant is unclear. In the current experiments, a commercially available FDA-approved Tisseel fibrin sealant containing a fibrinolysis inhibitor in the system was utilized. Despite this, more bone formation was still seen in the fibrin sealant group. Theoretically, a blood clot is analogous to a hematoma; it not only contains fibrin gel scaffold, but also serum/plasma and a variety of different blood cells as well as circulating growth factors and cytokines. In the setting of bone regeneration, blood clots mimic the natural healing environment, including inflammation, which results in white cell scavenging of the tissue damage debris, cell proliferation of regenerative cells including mesenchymal cells and vascular cells, mesenchyme condensation, chondrogenic differentiation and endochondral bone formation, and, finally, bone remodeling [40]. In the current study, the lenti-BMP2/GFP-transduced hMDSCs served as regenerative cells as well as a bone growth factor delivery system which orchestrates host blood cells in the bone regeneration process. However, a blood clot also has a large number of red blood cells, which normally function as oxygen carriers to systemic cells of all tissues. Once red blood cells are trapped in the clot, they are degraded and cleared by macrophages and, therefore, they may pose a burden for macrophages and delay their transition from M1 to M2 macrophages, a process which facilitates tissue repair and results in less bone formation. Furthermore, when surgery was performed, surgical site bleeding and hemostasis was achieved and rinsed with PBS before implanting the hMDSCs with fibrin sealant or autologous blood clot in an effort not to interfere with the implanted scaffold. Finally, the iron released from the degraded red blood cells may also have affected the initial hMDSCs survival after transplantation and again potentially interfering with the amount of bone regenerated. It is also possible that hMDSCs did not survive as well in the blood clot scaffold than in fibrin sealant due to the reasons described above. Therefore, the release of less BMP2 to induce host cell-mediated bone regeneration may have subsequently resulted in less new bone formation.

Finally, GFP staining was performed and no differences between the blood clot and fibrin sealant groups was observed. No significant difference for osteoclasts (TRAP^+^) and osteoblasts (OSX^+^) in the new regenerated bone between the groups were observed. It is believed that during the bone regeneration process, as long as hMDSCs survive, they can make similar contributions to the new regenerated bone despite a decreased quantity. Hence, autologous blood clot scaffolds may be more suitable as delivery vehicles for growth factors rather than cells.

## 5. Conclusions

In summary, autologous blood clots can serve as a safe scaffold for bone regeneration in critical-sized bone defects; however, the newly formed bone volume was significantly less in the BC group than in the FS group. Although many reports have shown blood clot scaffolds can promote tissue repair, fibrin sealant are more effective in promoting hMDSCs-mediated bone regeneration in a critical-sized calvarial defect model. Blood clots, although convenient, autologous, and safe as scaffolds for bone tissue engineering, may be more suitable as delivery vehicles for bone morphogenetic factors in the setting of bone regeneration. If using stem cells, fibrin sealant is more advantageous and leads to more bone formation without the negative effects on the donor and host cells.

## Figures and Tables

**Table 1:** Clot formation test conditions and results.

Test Conditions	Test Sites	Blood Draw Method	Whole Blood Volume (µL)	PBS Volume (µL)	Clot Formation Time (Formed Gel) in Minutes
1	1.5 mL Eppendorf tube	Heart puncture	40	20	Not formed clot in 20 min
2	1.5 mL Eppendorf tube	Heart puncture	45	15	Not formed clot in 20 min
3	1.5 mL Eppendorf tube	Heart puncture	50	20	Not formed clot in 20 min
4	1.5 mL Eppendorf tube	Heart puncture	50	0	Not formed clot in 20 min
5	Calvarial bone defect	Retro-orbital capillary	50	10	Formed clot in 5 min
6	Calvarial bone defect	Retro-orbital capillary	50	20	Formed clot in 5 min
7	Calvarial bone defect	Retro-orbital capillary	50	0	Formed clot in 5 min

## Data Availability

Original data will be available upon request.

## Supplementary Materials

The following are available online at https://www.mdpi.com/article/10.3390/biomedicines9080983/s1, Figure S1: Representative 3D evaluation of new bone volume in critical-sized bone defects. Contours were drawn manually and by the morph function. New bone volume was computed automatically by including every contoured area in each slice. We chose Gauss sigma = 0.8, Gauss support = 1, and lower threshold = 163. The threshold determines the bone volume and other microarchitecture parameters.
