# Supplementary material for: Comparison of Autologous Blood Clots with Fibrin Sealant as Scaffolds for Promoting Human Muscle-Derived Stem Cell-Mediated Bone Regeneration
Source: Biomedicines. 2021 Aug 9;9(8):983. doi: 10.3390/biomedicines9080983 (PMC8391974; doi:10.3390/biomedicines9080983)
Supplement: Supplementary file 1 [file biomedicines-09-00983-s001.zip › biomedicines-1299958-supplementary.pdf]

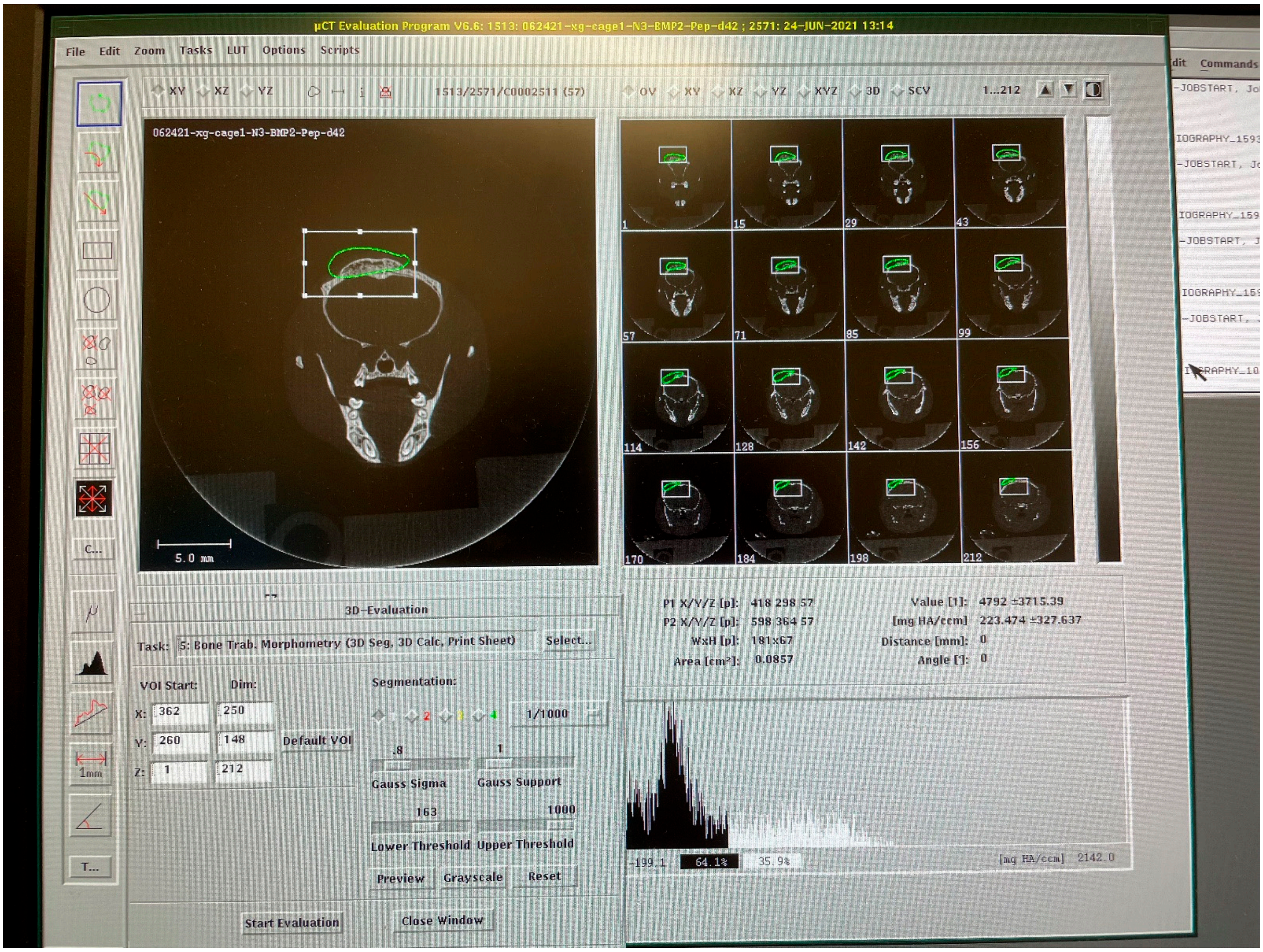


**Figure S1.** Representative 3D evaluation of new bone volume in critical sized bone defect. Contours were drawn manually and by morph function. New bone volume was covered automatically by the including every contoured area in each slice. We chose Gauss sigma = 0.8, Gauss support = 1 and lower threshold = 163. Threshold determines the bone volume and other microarchitecture parameters.
